# Adding biomarker change information to the kidney failure risk equation improves predictive ability for dialysis dependency in eGFR <30 ml/min/1.73 m^2^

**DOI:** 10.1093/ckj/sfae321

**Published:** 2024-10-24

**Authors:** Akira Okada, Shotaro Aso, Kayo Ikeda Kurakawa, Reiko Inoue, Hideaki Watanabe, Yusuke Sasabuchi, Toshimasa Yamauchi, Hideo Yasunaga, Takashi Kadowaki, Satoko Yamaguchi, Masaomi Nangaku

**Affiliations:** Department of Prevention of Diabetes and Lifestyle-Related Diseases, Graduate School of Medicine, The University of Tokyo, Tokyo, Japan; Department of Real-world Evidence, Graduate School of Medicine, The University of Tokyo, Tokyo, Japan; Department of Prevention of Diabetes and Lifestyle-Related Diseases, Graduate School of Medicine, The University of Tokyo, Tokyo, Japan; Department of Prevention of Diabetes and Lifestyle-Related Diseases, Graduate School of Medicine, The University of Tokyo, Tokyo, Japan; Department of Clinical Epidemiology and Health Economics, The University of Tokyo, Tokyo, Japan; Department of Real-world Evidence, Graduate School of Medicine, The University of Tokyo, Tokyo, Japan; Department of Diabetes and Metabolic Diseases, Graduate School of Medicine, The University of Tokyo, Tokyo, Japan; Department of Clinical Epidemiology and Health Economics, The University of Tokyo, Tokyo, Japan; Department of Prevention of Diabetes and Lifestyle-Related Diseases, Graduate School of Medicine, The University of Tokyo, Tokyo, Japan; Department of Diabetes and Metabolic Diseases, Graduate School of Medicine, The University of Tokyo, Tokyo, Japan; Toranomon Hospital, Tokyo, Japan; Department of Prevention of Diabetes and Lifestyle-Related Diseases, Graduate School of Medicine, The University of Tokyo, Tokyo, Japan; Division of Nephrology and Endocrinology, Graduate School of Medicine, The University of Tokyo, Tokyo, Japan

**Keywords:** biomarker, clinical epidemiology, dialysis dependency, kidney failure risk equation, prediction

## Abstract

**Background:**

Although the kidney failure risk equation (KFRE), a well-known predictive model for predicting dialysis dependency, is useful, it remains unclear whether the addition of biomarker changes to the KFRE model in patients with an estimated glomerular filtration rate (eGFR) <30 ml/min/1.73 m^2^ will improve its predictive value.

**Methods:**

We retrospectively identified adults with eGFR <30 ml/min/1.73 m^2^ without dialysis dependency, and available health checkup data for two successive years using a large Japanese claims database (DeSC, Tokyo, Japan). We dichotomized the entire population into a training set (50%) and a validation set (the other half). To assess the incremental value in the predictive ability for dialysis dependency by the addition of changes in eGFR and proteinuria, we calculated the difference in the C-statistics and net reclassification index (NRI).

**Results:**

We identified 4499 individuals and observed 422 individuals (incidence of 45.2 per 1000 person-years) who developed dialysis dependency during the observation period (9343 person-years). Adding biomarker changes to the KFRE model improved C-statistics from 0.862 to 0.921, with an improvement of 0.060 (95% confidence intervals (CI) of 0.043–0.076, *P* < .001). The corresponding NRI was 0.773 (95% CI: 0.637–0.908), with an NRI for events of 0.544 (95% CI of 0.415–0.672) and NRI for non-events of 0.229 (95% CI of 0.186–0.272).

**Conclusions:**

The KFRE model was improved by incorporating yearly changes in its components. The added information may help clinicians identify high-risk individuals and improve their care.

## Graphical Abstract

**Graphical Abstract:**
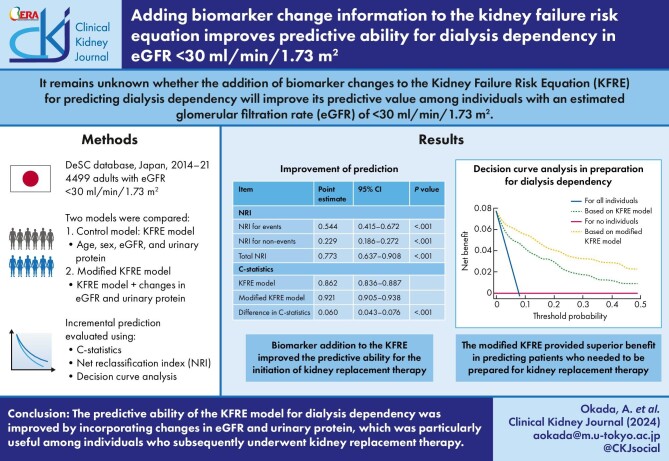

KEY LEARNING POINTS **What was known**:

- Individuals with an estimated glomerular filtration rate of <30 ml/min/1.73 m^2^ are at a high risk for kidney failure with dialysis dependency, and several models have been developed to predict the transition to dialysis dependency.
- The kidney failure risk equation has been useful for predicting the initiation of kidney replacement therapy.
- It remains unknown whether the addition of biomarker changes to the model will improve its predictive value.

**This study adds**:

- The addition of changes in eGFR and proteinuria improved the predictive ability of the kidney failure risk equation for the initiation of kidney replacement therapy.
- Analyses using decision curve analysis suggested that the modified model potentially improved clinical utility compared with the original kidney failure risk equation.

**Potential impact**:

- This model will help prepare adequate care, advance care planning, and follow-up among individuals at a high risk for kidney failure with dialysis dependency.

## INTRODUCTION

Chronic kidney disease (CKD), a noncommunicable disease, has become a global problem as its prevalence increases due to societal aging [1]. Although the age-standardized prevalence of CKD has remained the same since 1997, the age-standardized incidence of kidney failure with dialysis dependency has increased by >40% [2]. Therefore, efforts should focus on managing individuals at imminent risk for kidney replacement therapy. Individuals with severe kidney dysfunction, namely those with an estimated glomerular filtration rate (eGFR) of <30 ml/min/1.73 m^2^, are among the high-risk populations [3]. Such populations should be carefully followed up because the inadequate timing of dialysis initiation may lead to increased mortality [4]. To facilitate appropriate patient care, the decision to prepare suitable patients for the development of dialysis dependency should be based on the findings of individualized risk assessment [5]. Therefore, constructing an accurate and individualized risk-predictive model for dialysis dependency may improve the prognosis of patients with severe kidney dysfunction [5].

Currently, only a few models are used to predict the progression to dialysis-dependent kidney failure in patients with kidney dysfunction [5–7]. Of these, the kidney failure risk equation (KFRE) is the most extensively used, and its predictive power has been validated in studies involving global populations [8–13]. The four-variable KFRE comprises age, sex, eGFR, and urinary albumin [9], and the simplicity of this model facilitated the incorporation in electronic medical record [14]. Furthermore, the KFRE can predict not only the progression to dialysis dependency, but also increased cost due to kidney care [15]. Therefore, KFRE was used for the selection of high-risk populations regarding clinical decision-making [16, 17]. Indeed, the use of the KFRE equation for referral to kidney specialist or decision-making in advanced CKD could improve patient care [18].

Although the KFRE model is widely used and well-validated, whether the observation of any changes in components can improve the model's ability to predict progression to kidney failure among individuals with an eGFR of <30 ml/min/1.73 m^2^ remains unclear. Considering that changes in urinary protein levels and eGFRs are useful in predicting CKD progression [19–23], examining whether the addition of such information will increase the predictive ability of KFRE is important. Improving the predictive ability of KFRE will contribute to the advancement of effective intervention strategies in individuals with severe kidney dysfunction.

This study aimed to assess the incremental predictive value of biomarker alterations in the KFRE model for predicting dialysis-dependent kidney failure. Moreover, we assessed whether changes in proteinuria levels, and eGFRs will enhance the predictive ability of the model.

## MATERIALS AND METHODS

### Study design and data source

This population-based cohort study was conducted using information retrieved from the DeSC administrative claims database (DeSC Healthcare Inc., Tokyo, Japan). The DeSC database contains claims data submitted to health insurers at clinics, hospitals, and pharmacies. It contains information on the entire Japanese population, including people across all ages, and the details of the DeSC database have been described previously [24].

For claims data, we used diagnoses recorded according to the International Classification of Diseases, 10th revision (ICD-10) codes. The DeSC database contains annual health checkup data for ∼30% of the population. The checkup data included individual demographics (e.g. height, weight, and blood pressure), results of clinical laboratory tests (e.g. serum creatinine and urinary protein semi-quantitative tests), and responses to a questionnaire-based survey on lifestyle factors (e.g. smoking and ischemic heart disease history).

### Inclusion and exclusion criteria

Using the DeSC database, individuals (i) with an eGFR of <30 ml/min/1.73 m^2^ during the health checkup performed between 2014 and 2021, and (ii) with data on proteinuria and serum creatinine levels obtained in two successive checkups and with a look-back period of 6 months before the first checkup, were included in the study (Fig. S1). Individuals undergoing dialysis or kidney transplantation before the second checkup were excluded.

This study was approved by the Institutional Review Board of the Graduate School of Medicine at the University of Tokyo (2021010NI). The requirement for informed consent was waived owing to the use of anonymized data.

### Study variables

The following data were obtained and used in the KFRE used in this study: age, sex, eGFR, and semi-quantitative proteinuria levels recorded as five levels (negative, trace, 1+, 2+, and 3+). We also calculated albuminuria predicted by the five-level proteinuria, sex, and comorbidities of diabetes and hypertension [9, 25]. The inclusion of predicted albuminuria in the KFRE model was validated, supporting its use in the calculation [9, 25]. Moreover, we obtained information on biomarkers such as low-density lipoprotein cholesterol and HbA1c, comorbid diabetes or hypertension, and smoking history. We also collected information on systolic blood pressure and history of cardiocerebrovascular disease, the addition of which to the four-variable KFRE leads to a six-variable KFRE [9]. We utilized data obtained during the first health checkup to prepare the four-variable KFRE. We also retrieved information on proteinuria levels and eGFR measured during both the first and second health checkup to monitor changes in the markers.

### Study outcome

The primary outcome was the development of dialysis dependency, defined as the earliest date of hemodialysis, peritoneal dialysis, or kidney transplantation. Individuals were followed from the second checkup until the earliest date of dialysis initiation, kidney transplant, or censoring.

### Statistical analyses

We summarized the background characteristics of eligible individuals based on whether the outcome was reached or not. Categorical variables were compared using the chi-square test, and continuous variables were compared using Student's *t*-test. We described an incidence rate per 1000 person-years for the development of dialysis dependency.

We divided the participants into two sets: a training set, comprising a randomly selected 50% of the participants, and a validation set, comprising the remaining 50%. This method was adopted to avoid overfitting [26].

To prepare the models used for predicting the development of dialysis dependency, we estimated the risk of kidney failure using flexible parametric survival models (the Royston–Parmar models) using the training set [27, 28]. We used this parametric model in the survival analysis, which is useful in evaluating the prediction for event development [27, 28], instead of a semi-parametric model (i.e. Cox regression). To assess the incremental predictive ability of biomarker changes, we constructed two models: one was the KFRE model, and the other used the KFRE model with the incorporation of changes in eGFR and proteinuria levels (modified KFRE model). In both the KFRE and modified KFRE models, age and eGFR were treated as continuous variables, sex was a binary variable (male or female), and urinary protein was a five-level categorical variable (negative, trace, 1+, 2+, and 3+). In the modified KFRE model, the annual change in eGFR was represented as a continuous variable, indicating the yearly slope of eGFR change (expressed as ml/min/1.73 m^2^ per year, calculated by dividing the eGFR change by the interval between the two checkups), and the change in proteinuria was represented as a binary variable, indicating whether the dipstick proteinuria results underwent no or worse change across the five proteinuria categories. We observed “no or worse” change instead of improvement in proteinuria levels to avoid potential ceiling effects because individuals with the best proteinuria category (i.e. negative) in the first checkup could not have a better proteinuria result in the second checkup. The same dataset was used for both models, which were recalibrated for our study population using coefficients derived from parametric regression within the training set. The performance of the two models was evaluated by comparing the predicted probabilities at the 2-year mark with the actual occurrence of events. This corresponds to the evaluation of the original 2-year KFRE model.

The predictive ability of these models was evaluated. To determine the predictive ability of the two different models in predicting the risk of kidney failure, two performance measures were used: (i) the C-statistics and the difference between the C-statistics of the KFRE and that of the modified KFRE model with 95% confidence intervals (CIs) calculated using the jackknife approach [29], and (ii) the net reclassification index (NRI) from the KFRE to the modified KFRE model. The NRI was further divided into NRI for events and NRI for non-events, based on the changes in sensitivity and specificity, respectively [30].

We also assessed the clinical utility of the two models in predicting individuals requiring preparation for the development of dialysis dependency within two years using decision curve analysis (DCA) [31]. The DCA evaluates the trade-offs between the advantages of true positives (preparation for the development of dialysis dependency followed by the induction of kidney replacement therapy) and the potential disadvantages associated with false positives (preparation for the development of dialysis dependency without the subsequent induction of kidney replacement therapy) for various threshold probabilities [32, 33]. Each model was compared in addition to the two default scenarios: preparing for dialysis dependency across all or no individuals (first and second scenarios, respectively). This method involves analysis of the model's discrimination and calibration abilities, thereby enabling the assessment of the model's potential impact on clinical decision-making. The first scenario assumes that physicians would anticipate dialysis dependency (i.e. requiring intensive and frequent follow-up visits) for all participants without making any specific prediction for dialysis dependency. In this scenario, while individuals who will develop dialysis dependency during a specific period, presumably constituting a small portion of the total population, would benefit, those who will not develop dialysis dependency during the period, accounting for the rest of the total population, would incur excessive cost without tangible benefit (i.e. cost-exhausting). Conversely, the second scenario presumes a case wherein physicians would not perform any interventions for dialysis dependency for anyone, which would result in no net benefit for all individuals since no preparation or cost would be incurred.

We conducted additional research to determine the cut-off value for the yearly eGFR slope to predict the development of dialysis dependency. We described the receiver operating characteristic curve showing the balance of sensitivity and specificity of the yearly eGFR slope. The cut-off was determined using the Youden index [34].

Additionally, we performed subgroup analyses stratified by age (cut-off age 75 years) and sex, and conducted three sensitivity analyses. First, we used the six-variable KFRE as the reference model, wherein the systolic blood pressure and cardiocerebrovascular disease history were added to the four-variable KFRE model (sensitivity analysis 1). This analysis assessed whether including changes in eGFR and proteinuria improved the KFRE model, even with adjustments for systolic blood pressure and cardiocerebrovascular disease history. Next, we used the predicted albuminuria levels instead of semi-quantitative results (sensitivity analysis 2). Finally, we included individuals with an eGFR of <45 ml/min/1.73 m^2^ instead of those with an eGFR of <30 ml/min/1.73 m^2^ (sensitivity analysis 3).

A two-sided *P* value <.05 was considered statistically significant. All statistical analyses were performed using Stata version 18 (StataCorp, College Station, TX, USA).

## RESULTS

### Baseline characteristics

**Figure 1:**
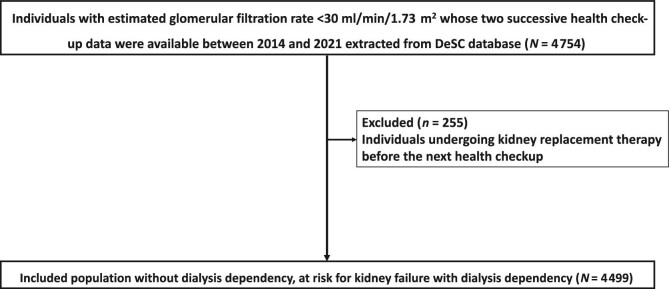
Flow chart of the selection process.

Among the population with health checkup data in the database, 4754 satisfied the inclusion criteria. We subsequently excluded 255 individuals who developed dialysis dependency before the second checkup, thereby including 4499 individuals in the analyses (Fig. 1).

The characteristics of the included individuals stratified by outcomes are summarized in Table 1. During follow-up (*N* = 9343 person-years at risk), 422 individuals (45.2 per 1000 person-years) developed kidney failure with dialysis dependency. Those who developed dialysis dependency during the follow-up period were more likely to be younger, male, have a higher systolic blood pressure, have a lower eGFR, have positive proteinuria by dipstick test, or have no history of cardiocerebrovascular disease (Table 1). In relation to alterations in biomarkers within the components of the KFRE, individuals who developed dialysis dependency exhibited a more intensive decrease in yearly change in eGFR. The characteristics of the populations belonging to the training and test sets were comparable (Table S1).

**Table 1:** Baseline characteristics of the eligible individuals.

		Without dialysis dependency development	With dialysis dependency development	
Variable	Category	*N* = 4077	*N* = 422	*P* value
Sex (male)		1938 (47.5%)	277 (65.6%)	<.001
Age (years)		78 (10)	69 (11)	<.001
Body mass index (kg/m^2^)		23.8 (3.7)	24.5 (4.5)	<.001
Body mass index category (kg/m^2^)	<18.5	237 (5.8%)	23 (5.5%)	<.001
	18.5–24.99	2466 (60.5%)	231 (54.7%)	
	25.00–29.99	1117 (27.4%)	120 (28.4%)	
	≥30.00	252 (6.2%)	48 (11.4%)	
	Missing	5 (0.1%)	0 (0.0%)	
Systolic blood pressure at initial checkup (mmHg)		132 (18)	138 (18)	<.001
Diastolic blood pressure at initial checkup (mmHg)		72 (12)	76 (12)	<.001
Smoking status	Non/past smoker	3476 (85.3%)	367 (87.0%)	<.001
	Current smoker	292 (7.2%)	50 (11.8%)	
	Missing	309 (7.6%)	5 (1.2%)	
Estimated glomerular filtration rate (ml/min/1.73 m^2^)		24.1 (5.3)	18.7 (5.9)	<.001
Estimated glomerular filtration rate category (ml/min/1.73 m^2^)	15–29	3787 (92.9%)	288 (68.2%)	<.001
	<15	290 (7.1%)	134 (31.8%)	
	Negative	1774 (43.5%)	36 (8.5%)	<.001
Proteinuria by dipstick test	Trace	528 (13.0%)	26 (6.2%)	
	+	769 (18.9%)	77 (18.2%)	
	2+	651 (16.0%)	152 (36.0%)	
	3+	355 (8.7%)	131 (31.0%)	
Predicted albumin-creatinine ratio (mg/gCr)		181.8 (346.3)	516.8 (502.0)	<.001
HbA1c (%)		5.9 (0.7)	6.0 (1.1)	<.001
	<5.7	2100 (51.5%)	210 (49.8%)	<.001
	5.7–6.4	1361 (33.4%)	115 (27.3%)	
HbA1c category (%)	6.5–7.9	500 (12.3%)	70 (16.6%)	
	≥8.0	68 (1.7%)	16 (3.8%)	
	Missing	48 (1.2%)	11 (2.6%)	
Low-density lipoprotein cholesterol (mg/dl)		109.1 (31.0)	108.0 (34.3)	.50
Comorbid diabetes		1784 (43.8%)	229 (54.3%)	<.001
Comorbid hypertension		3829 (93.9%)	412 (97.6%)	.002
History of cardiocerebrovascular disease	Without cardiocerebrovascular disease history	2026 (49.7%)	238 (56.4%)	.009
	With cardiocerebrovascular disease history	2051 (50.3%)	184 (43.6%)	
Yearly change in estimated glomerular filtration rate (mL/min/1.73m^2^/year)		2.1 (11.8)	−4.2 (4.2)	<.001
No or worse change in proteinuria level		1141 (28.0%)	131 (31.0%)	.18

Data are presented as the means (standard deviations) for continuous measures and *N* (%) for categorical measures.

*Body mass index, HbA1c, and low-density lipoprotein cholesterol were summarized after excluding 5, 59, and 10 patients without the corresponding information, respectively.

### Discrimination in the prediction of dialysis dependency

The hazard ratios of the KFRE and the modified KFRE models are listed in Table S2. The hazard ratios for dialysis-dependent kidney failure among the components of the KFRE model were similar. The yearly eGFR slope was associated with dialysis dependency (hazard ratio 1.30 for 1 ml/min/1.73 m^2^ decrease per year, 95% CI: 1.26–1.35), and no improvement in proteinuria levels was also associated with dialysis dependency (hazard ratio: 2.86, 95% CI: 2.02–4.04). Based on the regression results, we constructed 2-year recalibrated KFRE and modified KFRE models (Table S3).

Table 2 summarizes the discrimination abilities of the two models. The KFRE yielded a C-statistic of 0.862 (95% CI: 0.836–0.887), whereas that for the modified KFRE model was 0.921 (95% CI: 0.905–0.938), with a difference of 0.060 (95% CI: 0.043–0.076; *P* < .001).

**Table 2:** Model diagnostics for the prediction of dialysis dependency.

Item	Point estimate	95% confidence interval			*P* value
NRI					
NRI for events	0.544	0.415	–	0.672	<.001
NRI for non-events	0.229	0.186	–	0.272	<.001
Total NRI	0.773	0.637	–	0.908	<.001
C-statistics					
KFRE model	0.862	0.836	–	0.887	
Modified KFRE model	0.921	0.905	–	0.938	
ΔC-statistics	0.060	0.043	–	0.076	<.001

### Net reclassification index and decision curve analysis

Compared with the KFRE, the total NRI in the modified KFRE model was 0.773 (95% CI: 0.637–0.908). The addition of biomarker changes yielded NRIs of 0.544 for events (95% CI: 0.415–0.672) and 0.229 for non-events (95% CI: 0.186–0.272).

**Figure 2:**
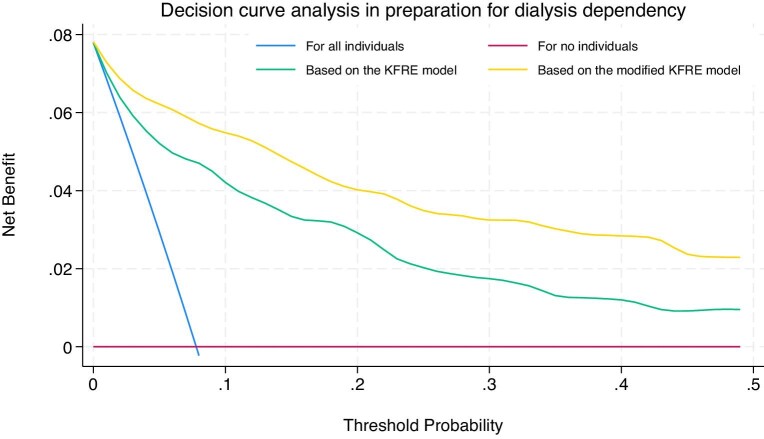
Decision curve for the dialysis dependency using the KFRE and modified KFRE models, with different threshold probabilities.

Based on the findings of the clinical effectiveness evaluation using DCA, the modified KFRE model provided the maximum benefit in predicting patients requiring preparation for dialysis dependency (Fig. 2). In particular, the new model provided a larger benefit than the KFRE in terms of clinical utility, suggesting an improvement in predicting patients requiring preparation for dialysis dependency after adding the changes of the two biomarkers to the KFRE.

### Cut-off for discrimination of development of dialysis dependency

The receiver operating characteristic curve based on the yearly eGFR slope is shown in Fig. S1. The optimal cut-off by the Youden index was −2.6 ml/min/1.73 m^2^/year. With this cut-off, the sensitivity was 65% and specificity was 74%.

### Stratified and sensitivity analyses

When stratified by age (<75 or ≥75 years) or sex (female or male), the results of the sensitivity analyses were similar (Table S4 and Figs S2 and S3), supporting the findings of the main analysis (Table S5 and Figs S4–S6).

## DISCUSSION

Using real-world data from a population-based database, we showed that the predictive ability of the KFRE model was improved by incorporating biomarker changes. The usefulness of these additional data was confirmed not only based on the NRI but also on the results of the DCA. Thus, the incorporation of biomarker changes in the KFRE is clinically useful for predicting the risk of dialysis dependency.

Although the KFRE has been used worldwide to predict kidney failure, studies aimed at improving its predictive ability, such as ours, are limited. Studies have shown conflicting results in the utility of additional information to the KFRE model in predicting dialysis dependency. For example, among patients with diabetic nephropathy and eGFR <60 ml/min/1.73 m^2^, the addition of the pathological information of kidney biopsy improved the risk for dialysis dependency calculated by the KFRE model, with an improvement of C-statistis by 0.0019 and NRI of 0.40 [35]. Another study revealed that among individuals with eGFR of 15–90 ml/min/1.73 m^2^, renal resistive index, an ultrasound maker of kidney, improved the KFRE model but the added model did not outperform the original KFRE model in the validation cohort [36]. Meanwhile, a recent study has shown that among individuals with eGFR of <60 ml/min/1.73 m^2^, the addition of information on an eGFR slope and cardiovascular comorbidity did not improve the KFRE model [37]. The discrepancy between the results of these studies and ours may be attributed to the different eGFR ranges of the included populations; our study included eGFR < 30 ml/min/1.73 m^2^; however, these studies included individuals with milder kidney dysfunction. The inclusion of this sample demographic may have led to a high baseline C-statistic (∼0.90), as shown in our sensitivity analysis 3 (Table S4), thereby making it difficult to detect a difference in C-statistics. Our inclusion criterion for eGFR (<30 ml/min/1.73 m^2^) could be justified because we focused on individuals at a high risk for dialysis dependency, wherein individualized decision-making is necessary. This study aimed to address the management strategies for this specific population.

The addition of changes in urinary protein levels and eGFR, both of which reportedly function as surrogate markers for CKD progression [19–23], improved the predictive ability of the KFRE in our study. The decrease in eGFR was shown to be useful in predicting kidney failure ∼10 years ago, whereas the incremental predictive value by incorporating information on the albuminuria/proteinuria change has been confirmed just recently [20–22]. A previous study also showed that incorporating the data on the ≥30% increase of albuminuria to that of the ≥30% decrease of eGFR after a multivariable adjustment resulted in the improvement in C-statistics (0.019) in the prediction of kidney failure [21]. Our study also detected the cut-off eGFR slope of −2.6 ml/min/1.73 m^2^ per year in predicting the development of dialysis dependency, which is consistent with a recent article showing that an eGFR slope of <−3 ml/min/1.73 m^2^ per year was consistently associated with increased risk for dialysis dependency while a slope of ≥−3 ml/min/1.73 m^2^ per year did not [37].

Beyond the KFRE models, recent studies have explored strategies for predicting dialysis dependency. For example, Tangri *et al.* developed a dynamic prediction model using time-varying biomarker changes, similar to our approach, achieving an integrated discrimination improvement of 0.73% by adding time-dependent covariates to baseline biomarker levels [38]. The most influential time-dependent variable was eGFR, followed by albuminuria, whereas albumin, phosphate, bicarbonate, and calcium levels were not significant determinants [38]. Additionally, recent studies have incorporated machine learning techniques to improve prediction accuracy. For example, Klamrowski *et al.* used a machine learning model to predict unplanned dialysis in patients at imminent risk of dialysis dependency [39]. Their random forest model incorporated age and time-varying trends in serum creatinine and albuminuria levels, achieving a C-statistic of up to 0.88 [39]. Another study, using the super learner machine learning algorithm [40], with information only on age, sex, eGFR, and albuminuria, outperformed the four-variable KFRE model in predicting dialysis dependency and death among patients with eGFR of 30–45 ml/min/1.73 m² [41]. Thus, these studies underscore the importance of time-dependent changes in serum creatinine and albuminuria as strong predictors of dialysis initiation and highlight the potential of advanced machine learning models to improve predictive accuracy.

Our study is novel in that the addition of biomarker changes may be useful in detecting individuals with subsequent kidney failure by measuring the NRI; moreover, DCA demonstrated the usefulness of the added biomarkers in several threshold probabilities. Routine follow-up, especially monitoring of changes in proteinuria levels and eGFR, should be performed in individuals with an eGFR of <30 ml/min/1.73 m^2^ and at a high risk predicted by the modified KFRE model. Monitoring serum creatinine and albuminuria/proteinuria routinely is recommended in guidelines [42, 43], and supported by recent studies that suggest that time-varying changes of both markers are important [38, 39].

The findings of the present study, aside from those regarding the predictive models, align with previous studies. The hazard ratios for kidney failure (Table S2) showed that younger age, male sex, decreased eGFR, and severe proteinuria were associated with progression to dialysis-dependent kidney failure in the KFRE model, which is consistent with the findings of previous studies [37, 44–46]. Other studies demonstrated that CKD progression decreased with age [44, 45], which is in line with our study reporting that advanced age had a negative association (hazard ratio of age <1 for the development of dialysis dependency) with CKD progression. Furthermore, the stratified and sensitivity analyses results revealed modification effects by sex or age, consistent with previous findings. The DCA curves in the age-stratified analysis showed that the clinical utility among individuals aged ≥75 years might be inferior to that among those aged <75 years. Although both groups had similar C-statistics, calibration among individuals aged ≥75 years may be less accurate, likely because the study did not account for the competing risk of death, as reported previously [47]. A possible effect modification by sex was also observed. Despite similar C-statistics for both sexes, the DCA curves suggested that calibration among females may be less accurate, likely due to the difference in the mean age (79.1 years in women vs 75.3 years in men). Further research on sex-based effect modification in the KFRE model is warranted.

Our study has several limitations. First, the absence of quantitative urinary protein/albumin data precluded their utilization in our analysis. However, considering that the semi-quantitative method as in our study was also shown to be useful in the KFRE model [37], the use of the semi-quantitative method may be justified. Second, this study used model development and validation cohorts in the database. Using the modified KFRE model in an external cohort may have resulted in a less pronounced effect from adding changes in eGFR and proteinuria. Therefore, the external validity, especially outside Japan, should be examined in future studies. Third, due to the retrospective nature of this study, other confounding factors, such as race, socioeconomic status, or frailty, may have been present. Moreover, the development of acute kidney injury, detailed medication history, or medication changes during the period might have affected the timing of kidney replacement therapy induction. Fourth, the original KFRE model already demonstrated high C-statistics; hence, the usefulness of the modified KFRE model for further improvement in predictive ability might be limited. Finally, we were unable to perform an analysis using the Fine and Gray model because information on death was only partially available for the population in the DeSC database.

In conclusion, this retrospective cohort study using a large-scale claims database revealed that the addition of biomarker changes was useful in increasing the predictive ability of the KFRE. Therefore, clinicians should pay attention to changes in eGFR and urinary protein to monitor CKD progression.

## Supplementary Material

sfae321_Supplemental_File

## Data Availability

The data underlying this article were provided by DeSC Healthcare, Inc. under license. Data will be shared on request to the corresponding author with the permission of DeSC Healthcare, Inc.
