# Supplementary material for: Adding biomarker change information to the kidney failure risk equation improves predictive ability for dialysis dependency in eGFR <30 ml/min/1.73 m2
Source: Clin Kidney J. 2024 Oct 24;17(11):sfae321. doi: 10.1093/ckj/sfae321 (PMC11574387; doi:10.1093/ckj/sfae321)

Table S1. Distributions of variables in the training and validation cohorts

Variable	Category	Training N=2,250	Validation N=2,249	p-value
Sex (male)		1,114 (49.5%)	1,101 (49.0%)	0.71
Age (years)		77 (10)	77 (10)	0.66
Body mass index (kg/m^2)		23.8 (3.9)	23.9 (3.8)	0.59
Body mass index category (kg/m^2)	<18.5	127 (5.6%)	133 (5.9%)	0.80
	18.5–24.99	1,365 (60.7%)	1,332 (59.2%)	
	25.00–29.99	610 (27.1%)	627 (27.9%)	
	≥30.00	146 (6.5%)	154 (6.8%)	
	Missing	2 (0.1%)	3 (0.1%)	
Systolic blood pressure at initial checkup (mmHg)		133 (18)	133 (18)	0.43
Diastolic blood pressure at initial checkup (mmHg)		72 (12)	72 (12)	0.87
Smoking status	Non/past smoker	1,914 (85.1%)	1,929 (85.8%)	0.45
	Current smoker	182 (8.1%)	160 (7.1%)	
	Missing	154 (6.8%)	160 (7.1%)	
Estimated glomerular filtration rate (mL/min/1.73m^2)		23.5 (5.6)	23.6 (5.6)	0.46
Estimated glomerular filtration rate category (mL/min/1.73m^2)	15–29	2,039 (90.6%)	2,036 (90.5%)	0.91
	<15	211 (9.4%)	213 (9.5%)	
	Negative	922 (41.0%)	888 (39.5%)	0.32
	Trace	259 (11.5%)	295 (13.1%)	
Proteinuria by dipstick test	+	409 (18.2%)	437 (19.4%)	
	2+	413 (18.4%)	390 (17.3%)	
	3+	247 (11.0%)	239 (10.6%)	
Predicted albumin-creatinine ratio (mg/gCr)		215.5 (376.0)	210.9 (377.1)	0.68
HbA1c (%)		5.9 (0.7)	5.9 (0.8)	0.65
HbA1c category (%)	<5.7	1,140 (50.7%)	1,170 (52.0%)	0.24
	5.7–6.4	753 (33.5%)	723 (32.1%)	
	6.5–7.9	289 (12.8%)	281 (12.5%)	
	≥8.0	34 (1.5%)	50 (2.2%)	
	Missing	34 (1.5%)	25 (1.1%)	
Low-density lipoprotein cholesterol (mg/dL)		109.4 (31.6)	108.5 (31.1)	0.36

Comorbid diabetes		1,012 (45.0%)	1,001 (44.5%)	0.75
Comorbid hypertension		2,116 (94.0%)	2,125 (94.5%)	0.52
History of cardiocerebrovascular disease	Without cardiocerebrovascular disease history	1,117 (49.6%)	1,147 (51.0%)	0.36
	With cardiocerebrovascular disease history	1,133 (50.4%)	1,102 (49.0%)	
Yearly change in estimated glomerular filtration rate (mL/min/1.73m^2/year)		1.5 (11.4)	1.5 (11.4)	0.88
No or worse change in proteinuria level		646 (28.7%)	626 (27.8%)	0.51
Days of follow-up		757.2 (485.4)	759.8 (489.1)	0.86
Dialysis dependency developed		216 (9.6%)	206 (9.2%)	0.61

Data are presented as the means (standard deviations) for continuous measures and N (%) for categorical measures.

*Body mass index, HbA1c, and low-density lipoprotein cholesterol were summarized after excluding 5, 59, and 10 patients without the corresponding information, respectively.

Table S2. Hazard ratios for kidney replacement therapy in the KFRE model and modified KFRE model

The KFRE model					
Variable	Category	Hazard ratio	95% confidence interval		
Age increase (10 years)		0.76	0.67	–	0.86
Male		1.53	1.15	–	2.03
eGFR		0.87	0.85	–	0.89
Urinary protein	Negative	Reference			
	Trace	3.44	1.67	–	7.07
	1+	7.80	4.28	–	14.22
	2+	16.80	9.57	–	29.48
	3+	23.66	13.25	–	42.26
The modified KFRE model					
Variable	Category	Hazard ratio	95% confidence interval		
Age increase (10 years)		0.79	0.69	–	0.90
Male		1.72	1.28	–	2.30
eGFR		0.78	0.76	–	0.81
Urinary protein	Negative	Reference			
	Trace	1.30	0.62	–	2.73
	1+	2.52	1.40	–	4.55
	2+	5.70	3.25	–	9.99
	3+	9.64	5.22	–	17.81
No improvement in urinary protein		2.86	2.02	–	4.04
Change in eGFR slope (per minus one)		1.30	1.26	–	1.35

KFRE, Kidney Failure Risk Equation; eGFR, estimated glomerular filtration rate

Table S3. Hazard ratios for kidney replacement therapy in the KFRE model and modified KFRE model

Two-year recalibrated KFRE model
1 – 0.9287 ^ exp (-0.2735 × (age/10 – 7.723) + 0.4260 × (male – 0.4923) – 0.1379 × (eGFR – 23.5785) (+ 1.2353 if trace proteinuria) (+ 2.0544 if proteinuria of "+") (+ 2.8212 if proteinuria of "2+") (+ 3.1637 if proteinuria of "3+"))
Two-year recalibrated modified KFRE model
1 – 0.9956 ^ exp (-0.2357 × (age/10 – 7.723) + 0.5414 × (male – 0.4923) – 0.2445 × (eGFR – 23.5785) (+ 0.2655 if trace proteinuria) (+ 0.9255 if proteinuria of "+") (+ 1.740 if proteinuria of "2+") (+ 2.2656 if proteinuria of "3+") + 0.2660 × eGFR yearly slope (+ 0.2655 if no improvement in proteinuria))
Original 2-year modified KFRE model (JAMA. 2016;315(2):164-74)
1 – 0.9750 ^ exp (-0.2201 × (age/10 – 7.036) + 0.2467 × (male – 0.5642) – 0.5567 × (eGFR/5 – 7.222) + 0.4510 × (logACR – 5.137))

Abbreviations: KFRE, Kidney Failure Risk Equation; eGFR, estimated glomerular filtration rate; ACR, albumin-creatinine ratio

Table S4. Model diagnostics for the prediction of kidney failure in analyses stratified age and sex.

Type of stratum	Item	Point estimate	95% confidence interval			P value
Age < 75 years	NRI					
N=1,512	NRI for events	0.507	0.354	–	0.660	<0.001
	NRI for non-events	0.194	0.116	–	0.273	<0.001
	Total NRI	0.701	0.530	–	0.873	<0.001
	C-statistics					
	KFRE model	0.811	0.773	–	0.848	
	Modified KFRE model	0.910	0.891	–	0.929	
	ΔC-statistics	0.099	0.070	–	0.129	<0.001
Age ≥ 75 years	NRI					
N=2,987	NRI for events	0.618	0.384	–	0.852	<0.001
	NRI for non-events	0.244	0.192	–	0.296	<0.001
	Total NRI	0.862	0.622	–	1.102	<0.001
	C-statistics					
	KFRE model	0.871	0.828	–	0.914	
	Modified KFRE model	0.915	0.885	–	0.945	
	ΔC-statistics	0.045	0.023	–	0.067	<0.001
Female	NRI					
N=2,284	NRI for events	0.508	0.272	–	0.743	<0.001
	NRI for non-events	0.272	0.213	–	0.332	<0.001
	Total NRI	0.780	0.537	–	1.023	<0.001
	C-statistics					
	KFRE model	0.840	0.793	–	0.886	
	Modified KFRE model	0.910	0.877	–	0.943	
	ΔC-statistics	0.070	0.039	–	0.102	<0.001
Male	NRI					
N=2,215	NRI for events	0.560	0.407	–	0.713	<0.001
	NRI for non-events	0.180	0.117	–	0.243	<0.001
	Total NRI	0.741	0.575	–	0.906	<0.001
	C-statistics					
	KFRE model	0.848	0.815	–	0.882	

	Modified KFRE model	0.913	0.891	–	0.934	
	ΔC-statistics	0.065	0.043	–	0.086	<0.001

KFRE, Kidney Failure Risk Equation; NRI, net reclassification index

Table S5. Model diagnostics for the prediction of dialysis dependency in sensitivity analyses.

Type of sensitivity analysis	Item	Point estimate	95% confidence interval			P value
Sensitivity analysis 1	NRI					
(Using the 6-variable KFRE model)	NRI for events	0.544	0.415	–	0.672	<0.001
	NRI for non-events	0.229	0.186	–	0.272	<0.001
	Total NRI	0.773	0.637	–	0.908	<0.001
	C-statistics					
	KFRE model	0.866	0.841	–	0.891	
	Modified KFRE model	0.922	0.906	–	0.939	
	ΔC-statistics	0.056	0.040	–	0.072	<0.001
Sensitivity analysis 2	NRI					
(Using predicted albuminuria levels)	NRI for events	0.544	0.415	–	0.672	<0.001
	NRI for non-events	0.229	0.186	–	0.272	<0.001
	Total NRI	0.773	0.637	–	0.908	<0.001
	C-statistics					
	KFRE model	0.869	0.844	–	0.893	
	Modified KFRE model	0.922	0.905	–	0.938	
	ΔC-statistics	0.053	0.039	–	0.067	<0.001
Sensitivity analysis 3	NRI					
(Individuals with eGFR < 45 ml/min/1.73 m^2)	NRI for events	0.646	0.541	–	0.750	<0.001
	NRI for non-events	0.238	0.224	–	0.253	<0.001
	Total NRI	0.884	0.779	–	0.990	<0.001
	C-statistics					
	KFRE model	0.934	0.917	–	0.950	
	Modified KFRE model	0.952	0.939	–	0.966	
	ΔC-statistics	0.019	0.011	–	0.026	<0.001

KFRE, Kidney Failure Risk Equation; NRI, net reclassification index; eGFR, estimated glomerular filtration rate

Figure S1. Overview of study design and model preparation.

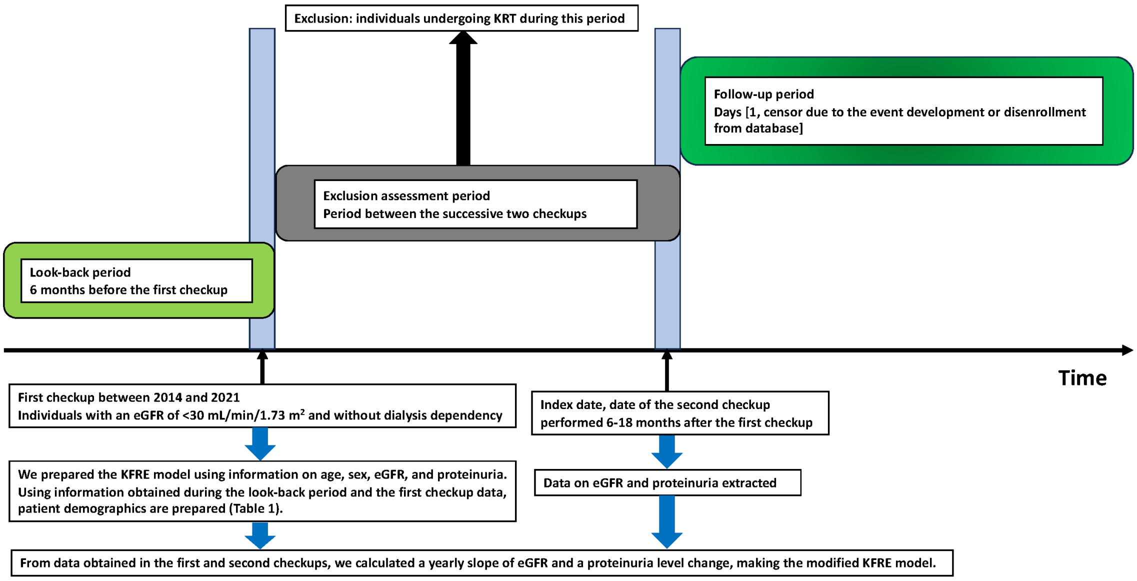

Abbreviations: KRT, kidney replacement therapy; eGFR, estimated glomerular filtration rate.

Figure S2. Receiver operating characteristic curve in the prediction of dialysis dependency development with use of yearly slopes of estimated glomerular filtration rate.

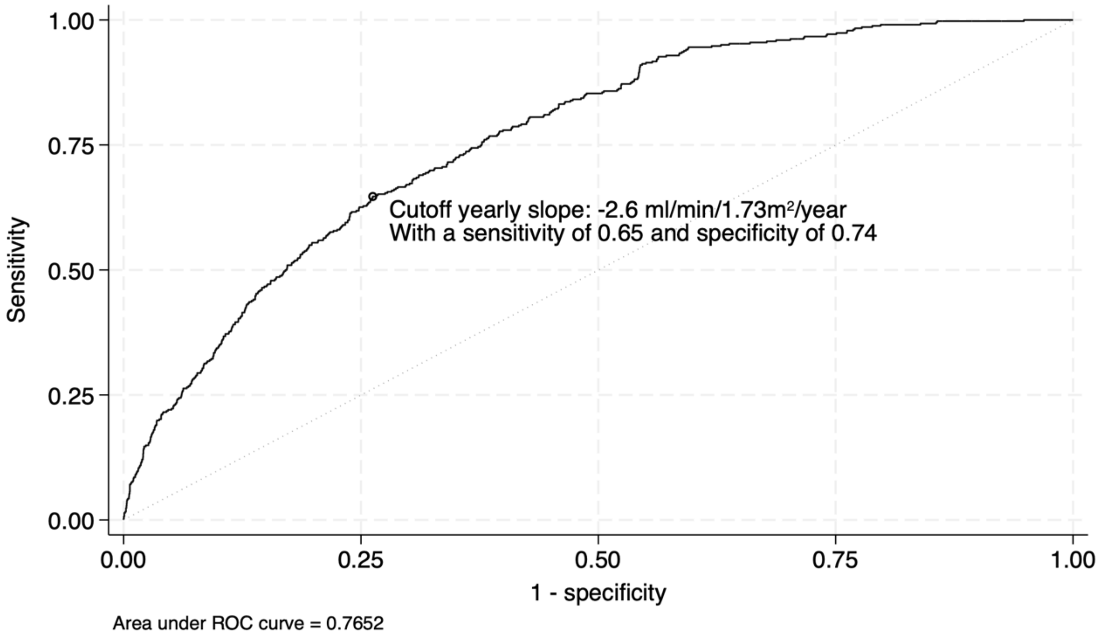

Figure S3. Decision curve for the dialysis-dependency prediction using the KFRE and modified KFRE models, with different threshold probabilities in the age-stratified analysis.

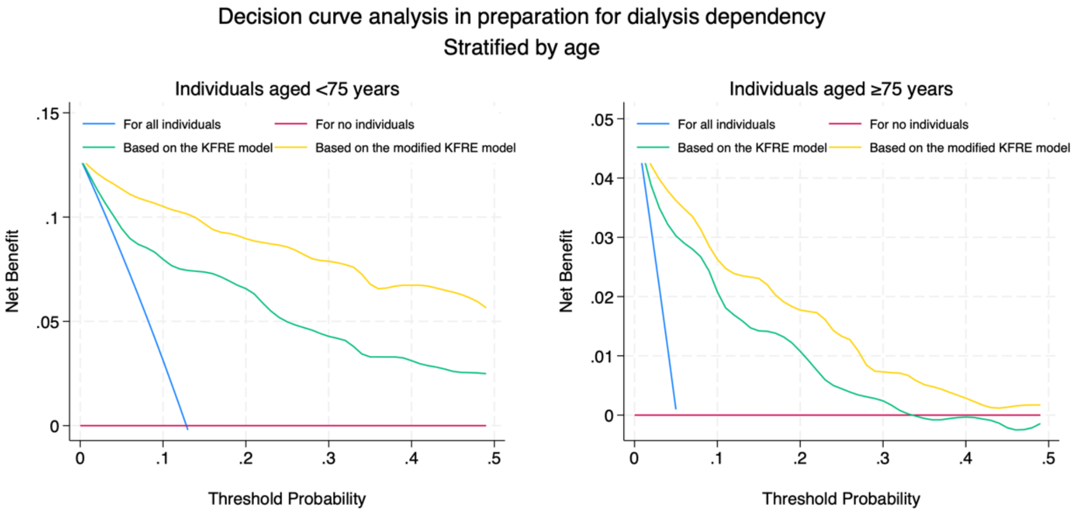

Figure S4. Decision curve for the dialysis-dependency prediction using the KFRE and modified KFRE models, with different threshold probabilities in the sex-stratified analysis.

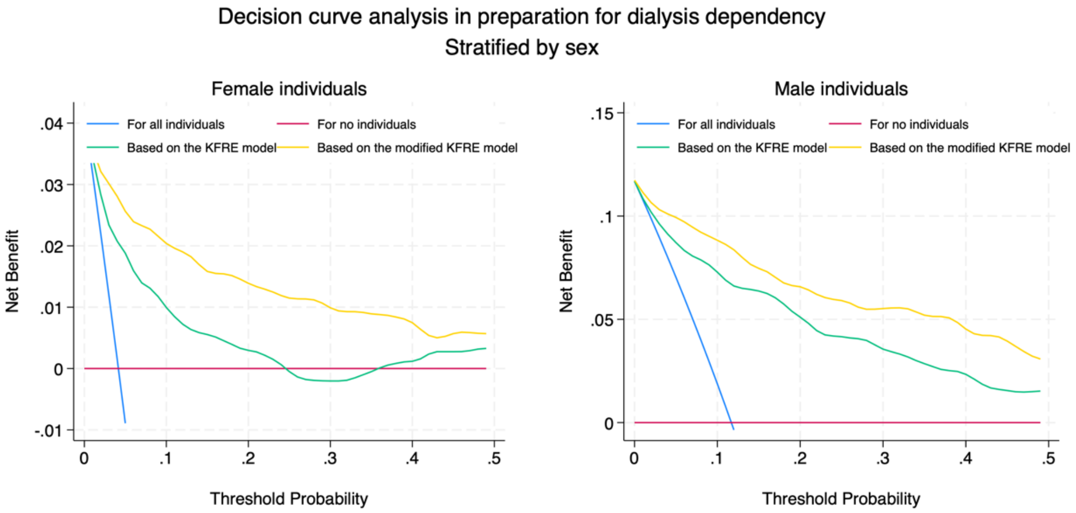

Figure S5 Decision curve for the dialysis-dependency prediction using the KFRE and modified KFRE models, with different threshold probabilities in Sensitivity analysis 1 (using the 6-variable instead of 4-variable KFRE model as the reference).

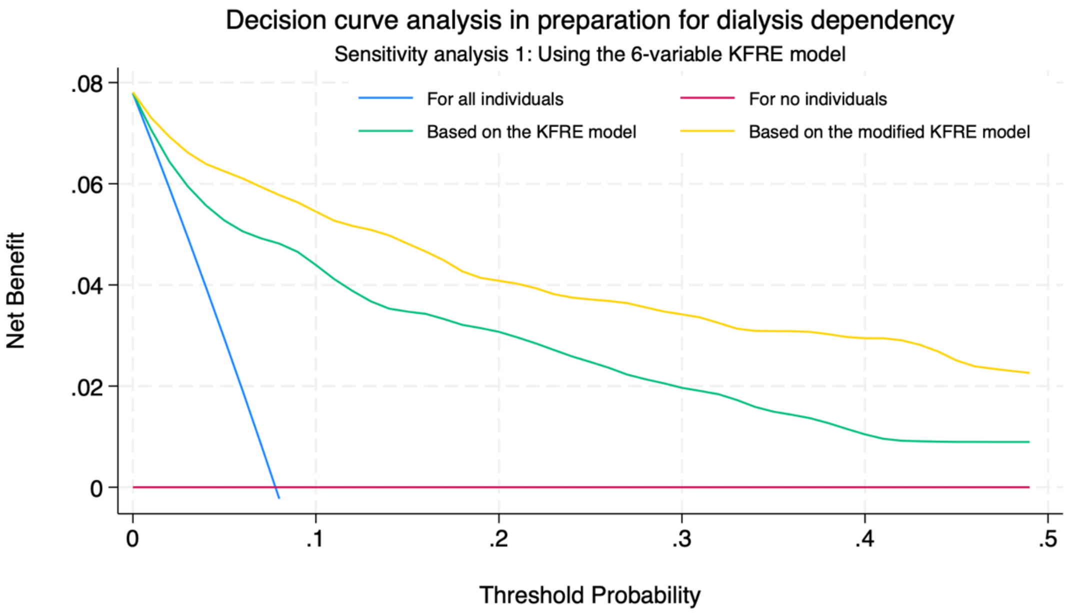

Figure S6. Decision curve for the dialysis-dependency prediction using the KFRE and modified KFRE models, with different threshold probabilities in Sensitivity analysis 2 (using the predicted albuminuria levels).

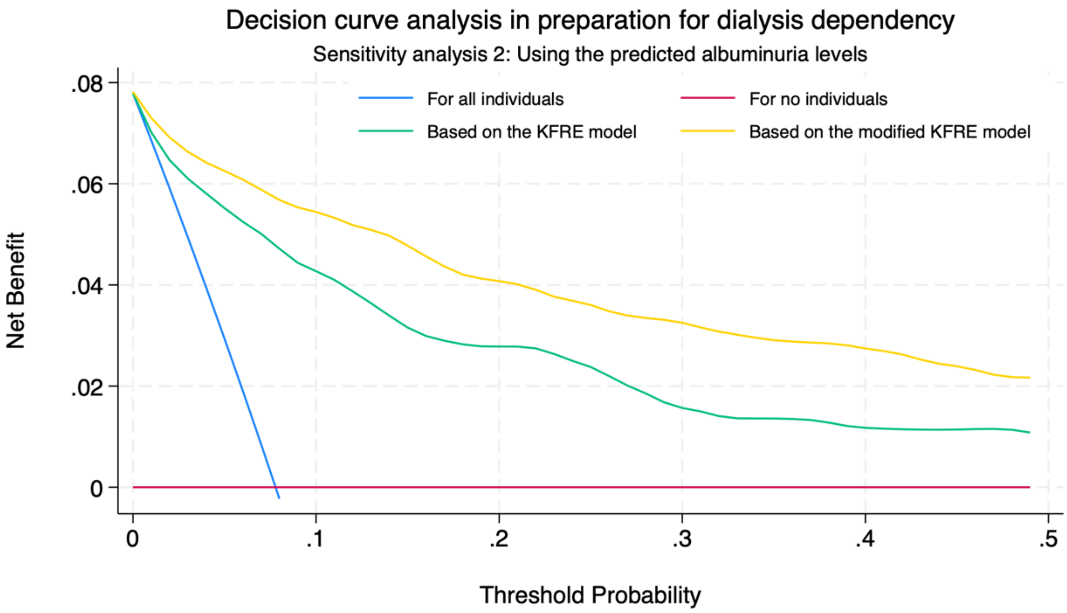

Figure S7. Decision curve for the dialysis-dependency prediction using the KFRE and modified KFRE models, with different threshold probabilities in Sensitivity analysis 3 (individuals with estimated glomerular filtration rate < 45 mL/min/1.73 m^2 instead of < 30 mL/min/1.73 m^2).

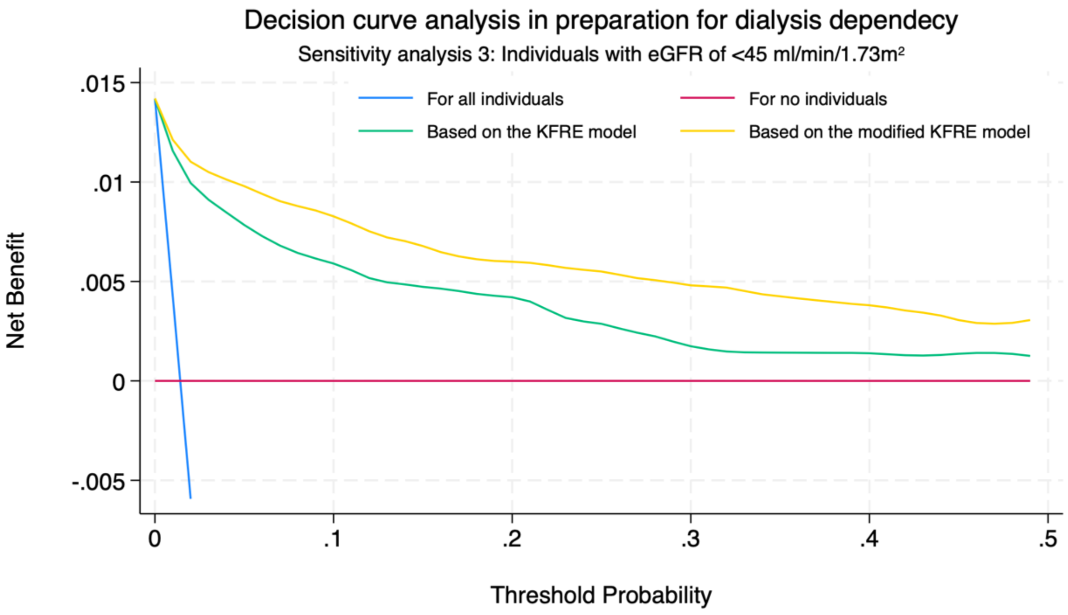

Supplement: sfae321_Supplemental_File [file sfae321_supplemental_file.pdf]
